# Lean-seafood intake increases urinary iodine concentrations and plasma selenium levels: a randomized controlled trial with crossover design

**DOI:** 10.1007/s00394-020-02366-2

**Published:** 2020-08-27

**Authors:** Jannike Øyen, Eli Kristin Aadland, Bjørn Liaset, Even Fjære, Lisbeth Dahl, Lise Madsen

**Affiliations:** 1 Institute of Marine Research, P.O. Box 1870 Nordnes, 5817 Bergen, Norway; 2 Department of Sport, Food and Natural Sciences, Western Norway University of Applied Science, Bergen, Norway; 3 Department of Biology, University of Copenhagen, Copenhagen, Denmark

**Keywords:** Arsenic, Iodine, Lean-seafood, RCT, Selenium

## Abstract

**Purpose:**

Iodine deficiency due to insufficient nutritional intake is a public health challenge in several European countries, including Norway. Lean-seafood has a high iodine and arsenic (As) content and is a good source of selenium (Se). Evidence of a direct effect of increased intake of lean-seafood on iodine status is limited. The main aims were to determine the iodine status at baseline and to investigate possible dietary effects on urinary iodine concentration (UIC) after intervention with lean-seafood versus non-seafood. Plasma Se, and plasma and urinary As concentrations were also measured.

**Methods:**

A randomized controlled crossover study comprising two 4 weeks experimental periods with two balanced diets varied in main proteins (60% of total dietary proteins) of lean-seafood and non-seafood, separated by a 5 week washout period.

**Results:**

Twenty participants (7 males, 13 females) were included and the mean ± SD age was 50.6 ± 15.3 years for all participants. Fasting UIC was median (25th, 75th percentile) 70 (38, 110) and 79 (49, 94) µg/L in the lean-seafood and non-seafood intervention at baseline, respectively. UIC increased after 4 weeks of the lean-seafood intervention to 135 (110, 278) µg/L, but not after the non-seafood intervention [58 (33, 91) µg/L] (*P* diet-effect < 0.001). Fasting plasma Se increased in the lean-seafood intervention and decreased in the non-seafood intervention (*P* diet-effect = 0.001). Fasting urinary and plasma As increased in the lean-seafood intervention and was unchanged in the non-seafood intervention (*P* diet-effect < 0.001).

**Conclusion:**

The participant’s UIC was below the recommended median (100 µg/L) at baseline, but increased sufficiently after a 4 week intervention with lean-seafood.

**Electronic supplementary material:**

The online version of this article (10.1007/s00394-020-02366-2) contains supplementary material, which is available to authorized users.

## Introduction

Iodine is essential for thyroid hormone production [1]. Suboptimal iodine status is a public health concern worldwide, and despite efforts to increase the dietary iodine intake, Europe is still the continent with the highest prevalence of iodine deficiency [2]. This is a particular concern for women that are pregnant and/or at childbearing age [3–7].

Dietary sources of iodine are limited. Hence, to cope with iodine insufficiency, iodized salt programmes are recommended [4, 8]. Such programmes have a high impact globally, but several European countries have not followed this strategy. The impact of iodized salt in Norway is limited as the amount of iodine in the iodized salt is low (5 mg iodine/kg salt), and the food industry is not allowed to use iodized salt. However, the cow feed has been fortified with iodine since the 1950s. Thus, milk and dairy products are the major sources of iodine in Norway [9, 10], and contributes with about 55% of the iodine intake [11]. Seawater fish and seafood are the food items where iodine is occurring at the highest concentrations [10, 12]. Although the concentration of iodine is higher in seafood than milk and dairy products there is lack of associations between intake of seafood and adequate UIC in Danish and Icelandic populations [13, 14]. However, a direct effect of increased intake of lean-seafood on UIC has to our knowledge been demonstrated in one intervention study [15]. Unlike iodized salt, intake of lean-seafood will also provide other components. Lean-seafood contains undesirable substances such as Hg [16], but also essential nutrients, such as selenium (Se), B vitamins, trace elements and some long chain n-3 polyunsaturated fatty acids [17]. Additionally, lean fish also provide high quality marine proteins and taurine [18].

UIC in spot urine samples is the recommended method to assess iodine status by World Health Organization (WHO), United Nations children’s fund (UNICEF) and Iodine Global Network (IGN). Importantly, however, UIC reflects the last 24 h of iodine intake. Hence, even if seafood is consumed 2–3 times a week as recommended, associations between seafood intake and UIC would necessarily be more difficult to reveal than associations with intake of items often consumed on a daily basis, such as milk and dairy. Thus, as lean-seafood is rarely consumed daily and UIC reflects the last day of iodine intake, the contribution of seafood for maintenance of sufficient iodine status may be underestimated in population-based studies.

To document the effect of lean-seafood on iodine status, we measured iodine in urine collected from participants in an intervention study, with a crossover design where lean-seafood was consumed every day and intake of milk and dairy were restricted. As lean-seafood also is the main contributor of dietary arsenic (As) and represents an important source for Se, we concurrently measured plasma levels of Se and As, as well as urinary As.

## Materials and methods

### Ethics statement

The study was registered at www.ClinicalTrials.gov (NCT01708681) and performed in accordance with the ethical standards of the regional committee on human experimentation. The protocol, informed consent and advertisements were approved by the Regional Committee for Medical and Health Research Ethics West, Norway (Reference # 2012/1084). All subjects signed a written informed consent.

### Study procedure and participants

This study is based on a previous performed RCT with crossover design where the primary outcome was the change in fasting and postprandial lipids from pre- to post-intervention during the two intervention periods. Experimental design and recruitment of participants are earlier described by Aadland et al. [19]. The participants were recruited during October and November 2012. The 1st intervention period was from January to February 2013 and the 2nd intervention period from April to May 2013.

Recruitment of participants, inclusion/exclusion criteria and their physical and clinical characteristics have been described [19]. Briefly, healthy, non-smoking, male and female Caucasian subjects, 18–65 years old were recruited in the great area of Bergen, Norway.

**Fig. 1:**
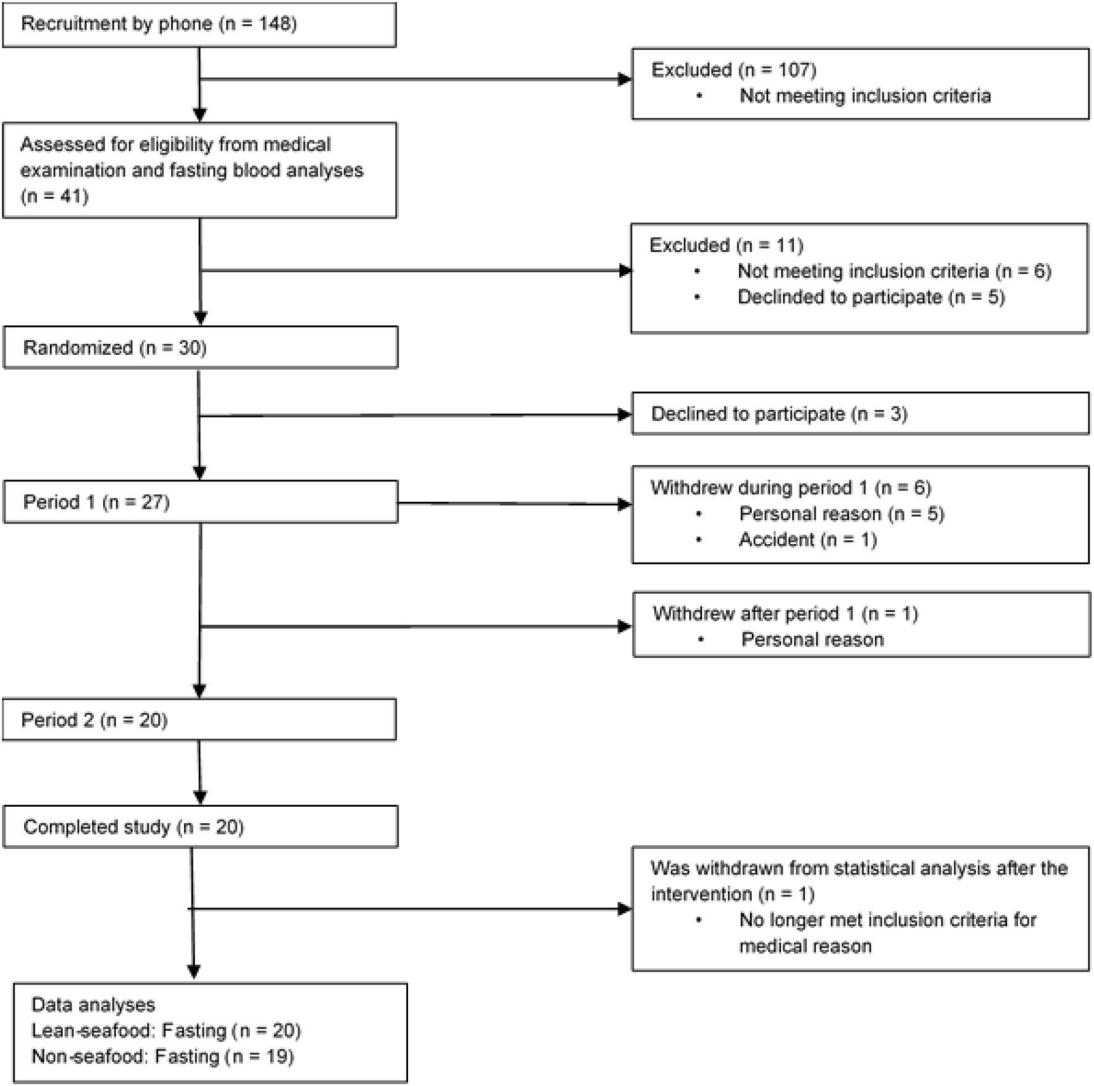
Consolidated flow chart for recruitment, randomization, and data sampling from the study participants

Based on data obtained from a pre-study visit [19], 30 subjects were invited to participate and 27 subjects accepted to start. Of these, 20 subjects completed period one (7 men and 13 women) and 19 subjects (7 men and 12 women) completed both periods (Fig. 1). No adverse events were reported during or after the intervention.

### Outcome measures and power calculation

The primary outcome in this RCT with crossover design was the change in fasting and postprandial lipids from pre- to post-intervention during the two intervention periods, and sample size was calculated based on the efficacy of fish protein to reduce plasma VLDL-TAG [19] and on the procedure described by Wellek and Blettner [20] for crossover design in clinical trials. A minimum of 16 subjects was needed to detect a treatment difference of ~ 25–30% in plasma VLDL-TAG at a probability level inferior to 0.05 and a power level corresponding to 80%. The primary outcome in the present paper was UIC, and secondary outcomes were plasma levels of Se and As, as well as urinary As.

### Experimental design

**Fig. 2:**
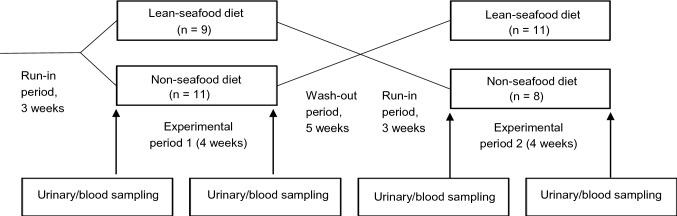
The crossover study design

As described previously [19], a controlled, randomized crossover study comprising two 4 weeks experimental periods, separated by a 5 week washout period was performed (Fig. 2). Prior to study start, the subjects were randomly allocated to start with one of the two dietary interventions. Randomization was performed by placing 30 pieces of papers, each piece with one of the participant’s identification number, and in an alternating order assigning the subjects to start with the lean-seafood or non-seafood diet by picking the paper pieces sequentially from the box. All participants were instructed to follow a diet in accordance with the Norwegian dietary recommendations [21] for 3 weeks (run-in period), with additional specifications to include a maximum of one fatty fish (salmon, trout, mackerel or herring) meal per week prior both experiments periods. During the last week of the run-in period and washout periods and throughout both experimental periods, the participants were instructed to avoid alcohol, chocolate or candy, industrial baked cakes or cookies, fast food, probiotics, and fish or fish oil supplements. They were also instructed to maintain their normal physical activity level during the run-in, experimental and washout periods. Fourteen subjects (6 men and 8 women) assigned to the lean-seafood diet consisting of lunch- and dinner meals with the 1st experimental period, whereas 13 subjects (4 men and 9 women) were assigned to a non-seafood diet. Compliance was assessed daily by an oral questionnaire. Only minor deviations occurred. Body mass was monitored and if necessary, dietary energy level adjusted to maintain a stable body weight (± 2 kg) in each experimental period.

### Diets

The experimental diets were prepared to meet the Norwegian nutrition recommendations [21], and formulated to meet energy the individual energy requirements for each participant as calculated from the self-reported food frequency questionnaire (FFQ) and using both the International (Harris–Benedict equation) [22] and the Nordic energy requirement references [23]. Six energy levels were established for each diet (7500, 8300, 9600, 10,900, 12,200 and 13,500 kJ/day). The experimental protein sources, covering 60% of total protein, the lean-seafood diet comprised cod, pollack, saithe and scallops, whereas the non-seafood diet comprised chicken breast filet without skin, lean beef, turkey breast filet without skin, pork, egg, low fat and skimmed milk (as ingredients in some of the meal recipes) and milk products (non-seafood diet). The remaining 40% of dietary protein was from vegetable and cereal sources. The experimental diets were balanced with equivalent amounts of dietary fibre, carbohydrates, protein, lipids, but not for cholesterol, which was higher for the non-seafood diet. Salt in both diets was restricted to a maximum of 5 g daily. Importantly, the participants were instructed to not drink milk during any of the interventions, and only small amounts of dairy products were included in the non-seafood diet. In the lean-seafood diet, the only dairy product was small amount of butter in the diets twice a week. To fulfil the Nordic recommendation for calcium and vitamin D, the participants received 750 mg calcium supplement during the lean-seafood intervention and 500 mg calcium supplement during the non-seafood intervention. For vitamin D, the participants daily received Vitamin D_3_ (10 µg or 400 IU) supplement during the lean-seafood intervention [23]. The two experimental diets were presented as 7 day rotating menus, and the composition of energy and nutrients was determined by the Norwegian Nutrient File database (“Kostholdsplanleggeren”) as shown in Table 1. The amino acid and the fatty acid composition, and examples of 1 day menus have been published previously [19]. Results from the analyses of Se in the lean-seafood and non-seafood intervention meals correspond to average 97 and 56 µg/day, respectively. The corresponding figures for As were 1094 and 13 µg/day, respectively.

**Table 1:** Mean ^a^composition of the 7 day experimental diets

	Lean-seafood	Non-seafood
Total energy (kJ)	10,882 ± 550	10,900 ± 452
Carbohydrates (% of energy)	52 ± 1	52 ± 1
Lipids (% of energy)	29 ± 1	29 ± 2
Protein (% of energy)	19 ± 1	19 ± 1
PUFA (g)	17 ± 5	17 ± 5
MUFA (g)	35 ± 5	35 ± 8
SFA (g)	25 ± 8	25 ± 3
EPA + DHA (g)	0.82	0.82^b^
Total fibre (g)	43 ± 4	43 ± 4
Iodine (µg)	828 ± 167	92 ± 29
Se (µg)	113 ± 18	54 ± 13

Data are mean ± SD

*DHA* docosahexaenoic acids, *EPA* eicosapentaenoic acid, *MUFA* monounsatured fatty acids, *PUFA* polyunsaturated fatty acids, *SFA* saturated fatty acids, *Se* selenium

^a^Mean of the 7 day menu cycle for the 10,900 kJ diet, as determined by the Norwegian nutrient file database (“Kostholdsplanleggeren”)

^b^The non-seafood dinner meals were supplemented with 3.3 g cod liver oil, containing 0.28 g EPA and 0.42 g DHA

The participants prepared breakfasts, evening meals and snacks at home using an approved food list adjusted to their experimental diet and energy level, whereas lunches were prepared at the Western Norway University of Applied Science and provided for the day after. During the weekdays, dinners were served at the Western Norway University of Applied Science, whereas weekend dinners were distributed on Fridays together with weekend and Monday lunches.

### Biochemical analyses

Fasting morning spot urine was collected at day 1 and at the last day of each experimental period. The urine was aliquoted and stored at − 80 °C prior to analyses. Blood was drawn from the antecubital vein at the clinic after an overnight fast at the first and the last day of each experimental period and EDTA plasma was prepared and stored at − 80 °C.

Urinary iodine and As concentrations [24], Se in plasma, and Se and As from the intervention meals [25, 26] were determined by Inductive Coupled Plasma Mass Spectrometry (ICP-MS) by standardized procedures at Institute of Marine Research. For the determination of total Se and As in plasma, samples were diluted to a final volume of 10 ml with deionized water. Prior to ICP-MS analyses [15], subsamples of 0.3 g were digested using 2.0 ml concentrated nitric acid (Merck, Darmstadt, Germany) and 0.50 ml 30% w/w hydrogen peroxide (Merck) in an Ethos Pro microwave system (Milestone, Sorisole, Italy). The technicians performing the analyses were blinded by giving the samples unique number codes.

### Statistical analyses

Continuous variables are expressed as mean with standard deviation (SD), and median with 25th, 75th percentile. All residuals were normally disturbed, checked using histograms and normal probability (Q–Q) plots. The paired t test was used to compare variables within the intervention groups. Mixed-model linear regression analyses were used to analyse possible diet-effects on the outcomes from pre- to post-intervention with the post-values as response and pre-values as covariate. The models were also adjusted for gender and/or age, but did not differ from the unadjusted analyses (data not shown).

Two-tailed *p* < 0.05 was considered statistically significant. Analyses were performed using the Statistical Package for the Social Sciences (SPSS^®^ Statistics Version 26).

## Results

### Characteristics of the participants

The mean ± SD age was 50.6 ± 15.3 years for all participants (*n* = 20), 49.7 ± 18.6 years for males and 51.0 ± 14.1 years for females. The average body weight and body mass index (BMI) was 86.3 ± 9.1 kg and 26.4 ± 3.0 kg/m^2^ in male, and 70.3 ± 7.0 kg, and 25.3 ± 3.0 kg/m^2^ in female participants. Other characteristics of the participants have been published previously [19].

### Urinary iodine concentration

**Fig. 3:**
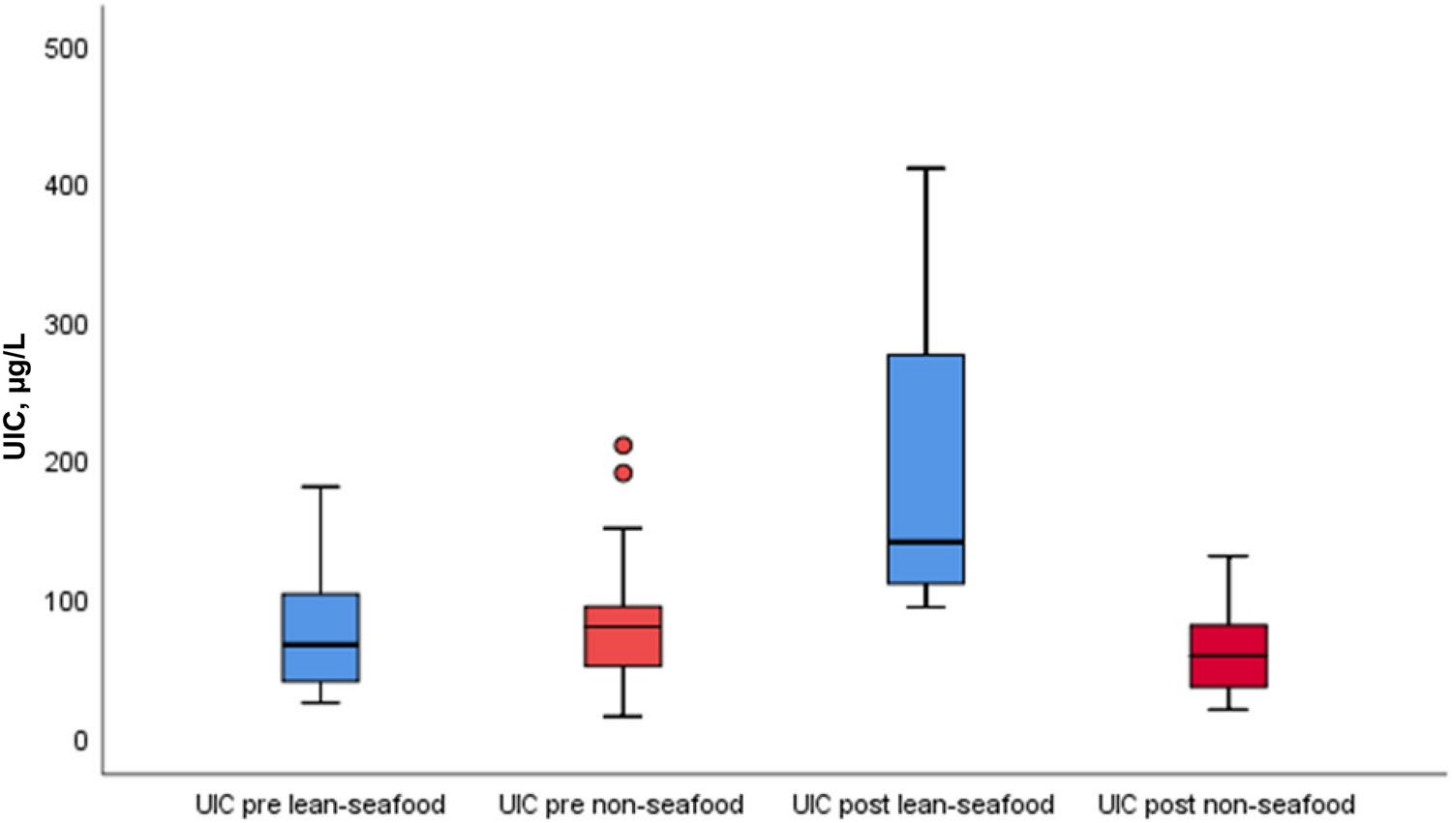
Pre-and post-intervention results of the UIC in µg/L within and between the lean-seafood (*n* = 20) and non-seafood (*n* = 19) interventions. The horizontal line through the box represents the median. The lower boundary of the box is the 25th percentile and the upper bounder is the 75th percentile. The smallest and largest observed values within the distribution are represented by the horizontal at either end of the box. Outliers are marked with circles. *P* for comparison of diet-effect between the intervention groups, mixed model regression analyses < 0.001. *UIC* urinary iodine concentration

Pre- and post-intervention results of UIC by median (25th, 75th percentile) within and between the interventions are given in Table 2 and Fig. 3. Median (25th, 75th percentile) UIC was 70 (38, 110) and 79 (49, 94) µg/L in the lean-seafood and non-seafood intervention at baseline, respectively. 4 weeks of the lean-seafood intervention increased UIC to 135 (110, 278) µg/L (*p* < 0.001), whereas UIC was not significantly changed after the non-seafood intervention [58 (33, 91) µg/L, *p* = 0.077], but a significant diet-effect was observed (*p* < 0.001) (Table 2, Fig. 3). Six (30%) of the participants in the lean-seafood intervention had UIC values ≥ 100 µg/L at baseline, and 18 (90%) at post-intervention. The corresponding figures in the non-seafood intervention were 4 (21%) at pre-intervention, and 3 (16%) at post-intervention.

**Table 2:** Urinary iodine and As concentrations and plasma Se and As concentrations in the subjects before and after 4 weeks interventions

Biochemical parameters (µg/L)	Lean-seafood intervention (*n* = 20)				Non-seafood intervention (*n* = 19)				
	Pre		Post		Pre		Post		
	*N*	Median (25th, 75th percentile)	Median (25th, 75th percentile)	*P*^a^	*N*	Median (25th, 75th percentile)	Median (25th, 75th percentile)	*P*^a^	*P*^b^
UIC	20	70 (38, 110)	135 (110, 278)	< 0.001	19	79 (49, 94)	58 (33, 91)	0.077	< 0.001
Plasma Se	19	70 (58, 73)	75 (68, 85)	< 0.001	17	69 (63, 75)	67 (60, 70)	0.015	0.001
Urinary As	20	62 (21, 180)	495 (300, 740)	0.002	19	48 (13, 96)	7 (5, 10)	0.059	< 0.001
Plasma As	19	5 (3, 8)	19 (9, 25)	0.001	17	4 (3, 11)	4 (3, 5)	0.651	< 0.001

*As* Arsenic, *Se* selenium, *UIC* urinary iodine concentration

^a^*P* for comparison within the intervention groups, paired sample *t *test

^b^*P* for comparison of diet effect between the intervention groups, mixed model regression analyses

### Plasma selenium

**Fig. 4:**
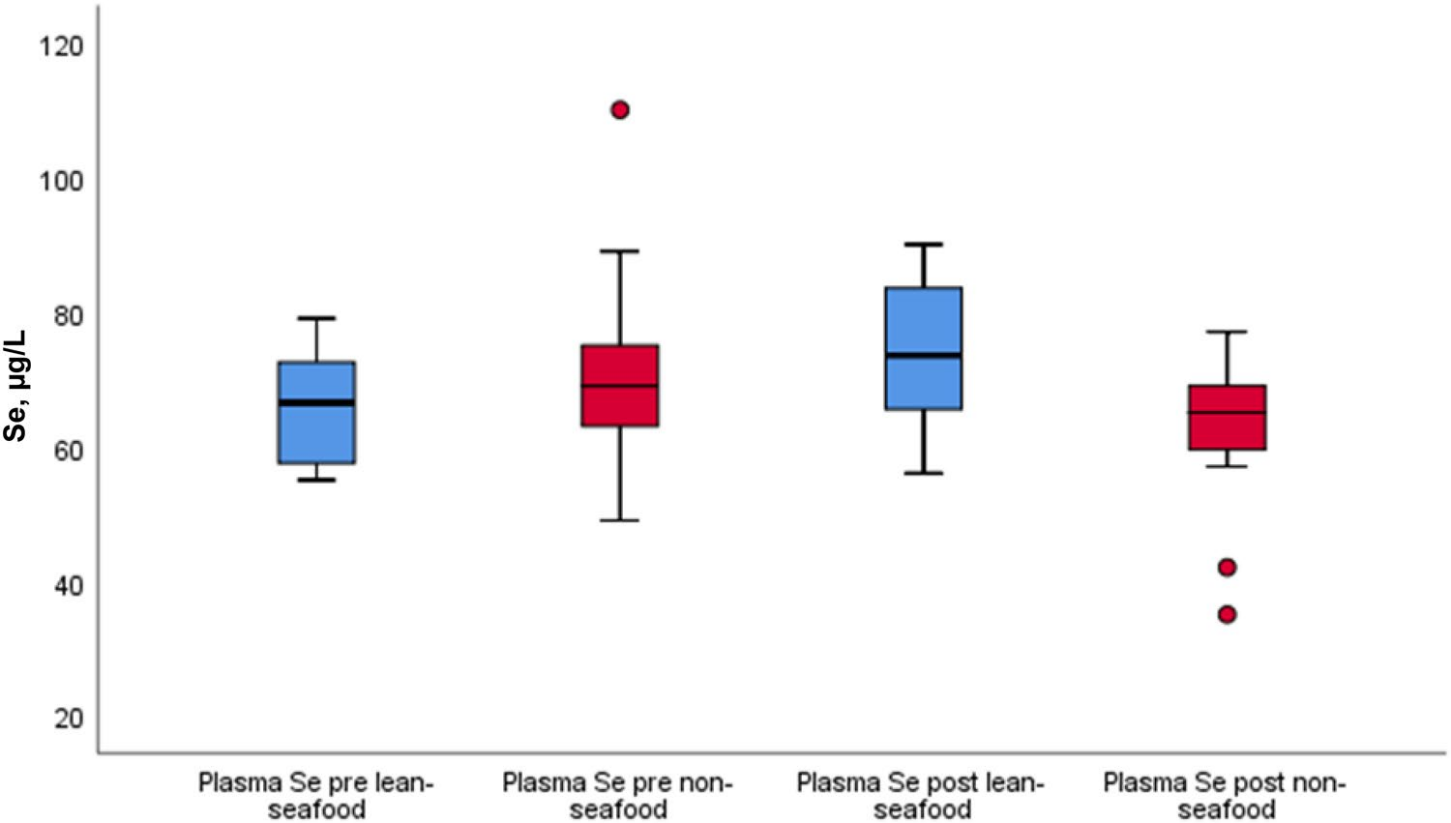
Pre-and post-intervention results of the plasma Se in µg/L within and between the lean-seafood (*n* = 19) and non-seafood (*n* = 17) interventions. The horizontal line through the box represents the median. The lower boundary of the box is the 25th percentile and the upper bounder is the 75th percentile. The smallest and largest observed values within the distribution are represented by the horizontal at either end of the box. Outliers are marked with circles. *P* for comparison of diet-effect between the intervention groups, mixed model regression analyses = 0.001. *Se* selenium

Pre- and post-intervention results of plasma Se by median (25th, 75th percentile) within and between the interventions are given in Table 2 and Fig. 4. Fasting plasma Se increased significantly from median (25th, 75th percentile) 70 (58, 73) µg/L to 75 (68, 85) µg/L (*p* < 0.001) in the lean-seafood intervention and decreased from 69 (63, 75) µg/L to 67 (60, 70) µg/L (*p* = 0.015) in the non-seafood intervention, and a significant diet-effect was observed (*p* = 0.001) (Table 2, Fig. 4).

### Urinary and plasma arsenic

**Fig. 5:**
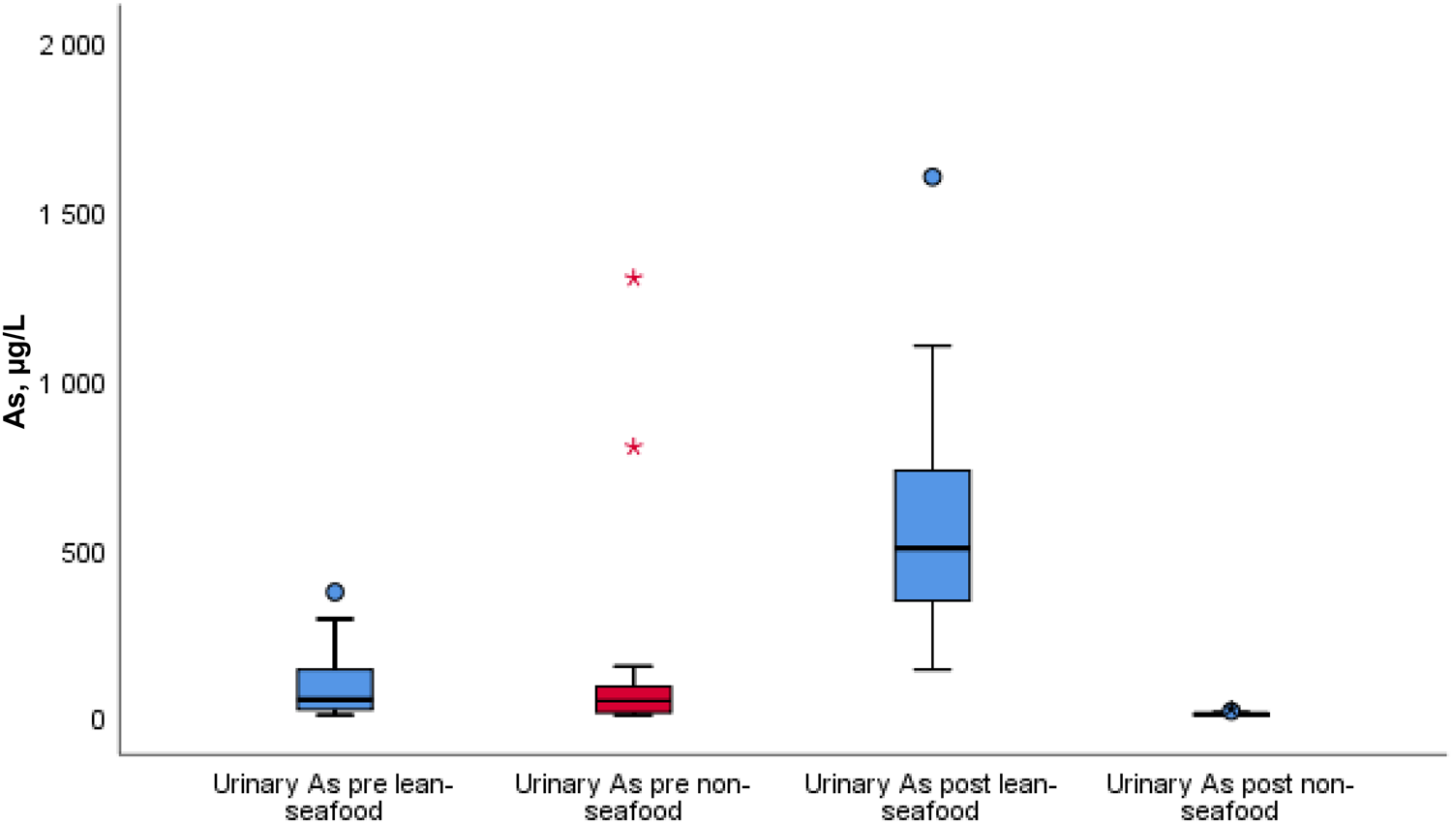
Pre-and post-intervention results of the urinary As in µg/L within and between the lean-seafood (*n* = 20) and non-seafood (*n* = 19) interventions. The horizontal line through the box represents the median. The lower boundary of the box is the 25th percentile and the upper bounder is the 75th percentile. The smallest and largest observed values within the distribution are represented by the horizontal at either end of the box. Outliers are marked with circles or asterisk (extreme points). *P* for comparison of diet-effect between the intervention groups, mixed model regression analyses < 0.001. *As* arsenic

**Fig. 6:**
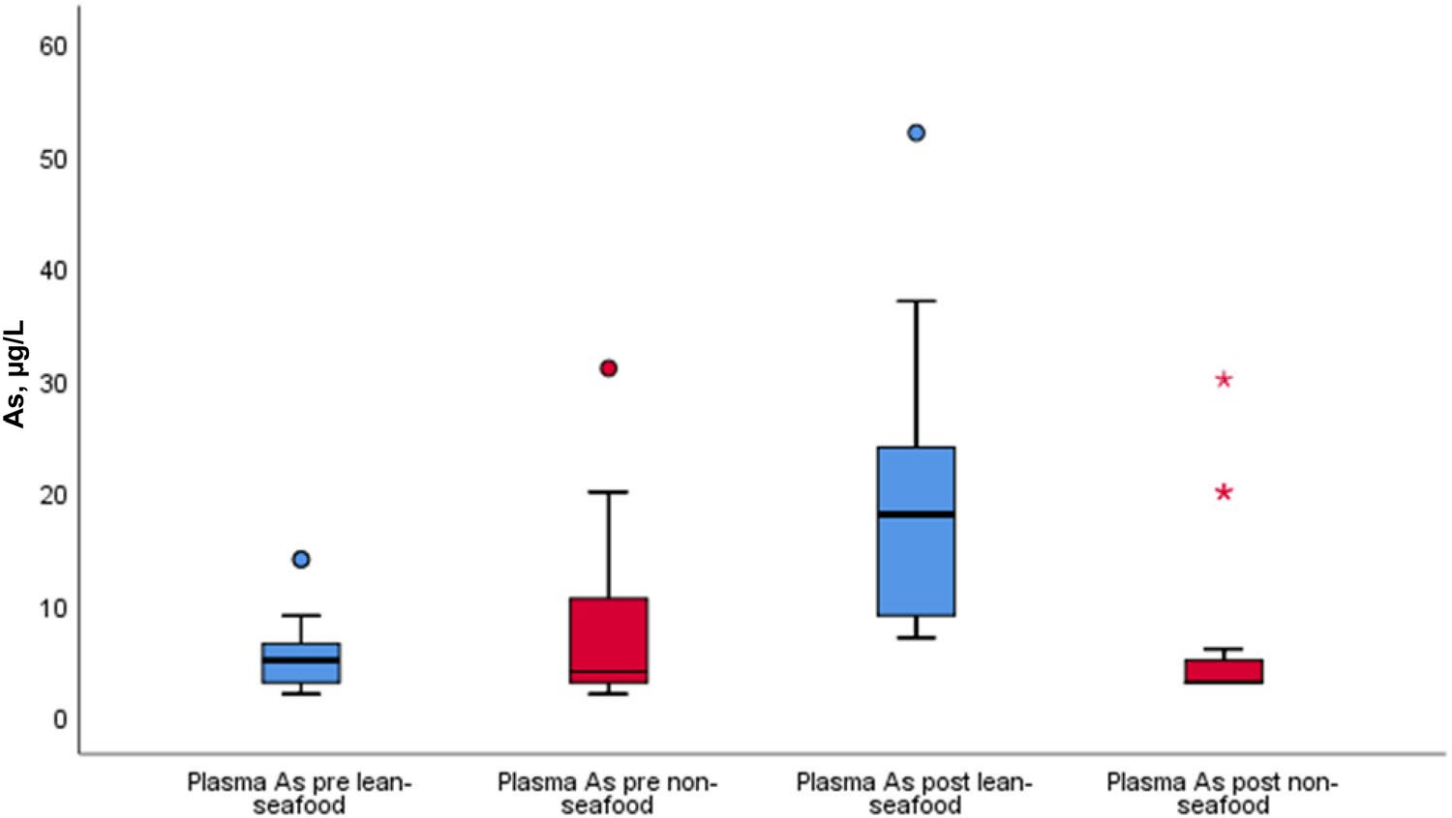
Pre-and post-intervention results of the plasma As in µg/L within and between the lean-seafood (*n* = 19) and non-seafood (*n* = 17) interventions. The horizontal line through the box represents the median. The lower boundary of the box is the 25th percentile and the upper bounder is the 75th percentile. The smallest and largest observed values within the distribution are represented by the horizontal at either end of the box. Outliers are marked with circles or asterisk (extreme points). *P* for comparison of diet-effect between the intervention groups, mixed model regression analyses < 0.001. *As* arsenic

Pre- and post-intervention results of fasting urinary and plasma As by median (25th, 75th percentile) within and between the interventions are given in Table 2 and Fig. 5 and 6. Urinary As increased significantly from median (25th, 75th percentile) 62 (21, 180) µg/L to 495 (300, 740) µg/L (*p* = 0.002) in the lean-seafood intervention and was unchanged in the non-seafood intervention [from 48 (13, 96) µg/L to 7 (5, 10) µg/L, *p* = 0.059], but a significant diet-effect was observed (*p* < 0.001) (Table 2, Fig. 5).

Plasma As increased significantly from median (25th, 75th percentile) 5 (3, 8) µg/L to 19 (9, 25) µg/L (*p* = 0.001) in the lean-seafood intervention and was unchanged in the non-seafood intervention [from 4 (3, 11) µg/L to 4 (3, 5) µg/L, *p* = 0.651], but a significant diet-effect was observed (*p* < 0.001) (Table 2, Fig. 6).

## Discussion

Lean-seafood has the highest natural iodine content, but evidence of a direct impact of increased intake of lean-seafood on UIC is limited. We therefore aimed to measure UIC after intervention with lean-seafood and non-seafood in an intervention study with crossover design. The participant’s UIC was below the recommended median at baseline, but after 4 weeks of the lean-seafood intervention median UIC was above 100 µg/L.

The present investigation demonstrated that a 28 day lean-seafood intervention increased median UIC by 65 µg/L, whereas the UIC was unchanged after the non-seafood intervention. Lean-seafood is a well-known source of iodine, but to our knowledge, this is the second intervention study directly demonstrating an effect of intake of lean-seafood on UIC. In a Norwegian 14 day semi-controlled study by Molin et al. [15], 38 participants were randomized into four groups for daily portions of 150 g cod, salmon, blue mussels or potato (control). The participants in the cod and blue mussel groups increased significantly their UIC from median 80–220 µg/L and 85–155 µg/L, respectively, compared to the control group with unchanged UIC from pre- to post-intervention (95 µg/L). Increases in UIC have also been demonstrated with cow milk [27], seaweed [28], and supplements [29]. Given the high iodine levels in lean-seafood, our result was expected. Further, the historically good iodine status in the Icelandic population has, at least in part, been attributed to a high intake of fish [30, 31]. Still, although seafood is suggested to contribute to iodine status, UIC has been associated with intake of milk and dairy, and not seafood, in population-based studies from for example Iceland [32], Norway [33, 34], and Italy [35]. Additional, in a Danish crossover study including a random sample of inhabitants living in Alborg and Copenhagen, estimated 24 h UIC increased with increased seafood intake in participants living in Alborg, but not in Copenhagen, whereas increased milk intake was associated with increased UIC in both cities. However, even though the intake of seafood was above 75 g/d in the group of Alborg participants who consumed most, the UIC was inadequate (median 71 µg/L), and adequate UIC was detected only in those living in Copenhagen who consumed more than two glasses of milk daily [14]. Unlike seafood, with a recommended intake of 2–3 times a week, milk and dairy are often consumed daily. As UIC reflects intake of iodine the last 24 h, associations between seafood and UIC may be more difficult to detect, and therefore the overall contribution of seafood to iodine status may be underestimated.

At baseline, the median UIC was below 100 µg/L and hence, iodine status in the participants comprising 20 healthy adult Norwegians may be considered suboptimal. The number of participants is small, but our finding that about 70% of the participants had UIC below 100 µg/L at study start corroborate the suggestion that iodine deficiency or sub-optimal iodine status is a re-emerging condition in Norway. For instance, recent studies have reported median UIC of 68 µg/L [3] and 85 µg/L [36] in pregnant women, and 75 µg/L in non-pregnant Norwegian women [37]. In addition, a small Norwegian cross-sectional study detected inadequate UIC and iodine intake in elderly (62 µg/L), pregnant women (84 µg/L), non-pregnant women of childbearing age (71 µg/L), and in vegans (46 µg/L) [38]. Due to introduction of iodine fortification of cow fodder in the 1950s and traditionally high intake of milk and dairy with subsequent high iodine concentrations, health authorities used to consider Norwegians to be iodine-replete [39]. The possible re-emergence of sub-optimal iodine levels in Norway may be linked to the recorded declining intake of both milk, dairy and seafood [40].

Concomitant with increased UIC after the lean-seafood intervention, we also observed increased urinary As levels and increased fasting plasma As and Se levels, similar to the findings by Molin et al. [15]. These results were expected, as lean-seafood will also provide other essential nutrients, such as Se, as well as undesirable substances. Seafood consumption is a predictor of elevated urinary As in several population studies and may be used as a biomarker [41]. Seafood and cereals appear to be the most important contributor to adequate blood Se in adolescent Icelandic girls [42]. The relatively high content of As and other heavy metals, such as mercury (Hg), may be of concern. However, in lean-seafood the major As form is the arsenobetaine, considered to be non-toxic [43]. Several risk benefit evaluations of seafood have concluded that the beneficial effects of the essential nutrients in seafood outweighs potential harmful effects of undesirable substances, including mercury [44–46]. In this respect, the content of Se may be of particular importance. First, low Se intake is reported in several countries [47], and lean finfish species are good dietary sources for Se [16, 48]. Second, Se is also known to antagonize the toxic effects of heavy metals, including Hg [49], and a molar ratio of Se: Hg above 1.0 is suggested to provide protection against MeHg toxicity in humans [50].

The strengths of this study include the crossover design and the balanced diets and accurate recordings of the seafood intake. The UIC measured in spot urine samples is the recommended method [4], but it reflects recent dietary intake and there is debated whether this is the best estimate of measuring iodine status. Thus, it is a strength that the participants consumed lean-seafood daily. Milk and dairy are major sources for iodine. Importantly, only very small amounts of dairy products were included in the non-seafood diet and all participants were instructed to not drink milk during any of the interventions.

This study has some limitations. The diets with 60% of dietary proteins from lean-seafood or non-seafood sources does not reflect a normal dietary protein intake for most people and the generalizability of our findings are thus limited. The comprehensive design limited the number of subjects willing to participate and led to a rather high drop-out (26%). This was, however, accounted for in the power analysis. The power calculation was, however, not based on UIC as the primary endpoint, but on cardiovascular lipid risk markers. Further, only Caucasians were included.

We conclude that 4 weeks of the lean-seafood intervention increased UIC from sub-optimal levels to adequate levels above 100 µg/L. Iodized salt programmes have had a high impact globally, but several European countries have not followed this strategy and a sub-optimal iodine level is evident among subpopulations in Europe. Unlike iodized salt, iodine rich food items, such as lean-seafood will provide essential nutrients and undesirable substances, such as Se and As.

## Electronic supplementary material

Below is the link to the electronic supplementary material.

Supplementary file1 (DOC 219 kb)

## Data Availability

Data (in anonymised form) used in the manuscript will be available upon request.

## Code availability

Statistical Package for the Social Sciences (SPSS^®^ Statistics Version 26).
